# A controller of robot constant force grinding based on proximal policy optimization algorithm

**DOI:** 10.1371/journal.pone.0319440

**Published:** 2025-05-07

**Authors:** Qichao Wang, Linlin Chen, Qun Sun, Chong Wang, Yanxia Wei

**Affiliations:** School of Mechanical and Automotive Engineering, Liaocheng University, Liaocheng, Shandong, China; Duke University, UNITED STATES OF AMERICA

## Abstract

In order to solve the problems of high dependence on the accuracy of environmental model and poor environmental adaptability of traditional control methods, the robot constant force grinding controller that based on proximal policy optimization was proposed. Training the controller model between grinding force difference and end-effector compensation displacement using the proximal policy optimization algorithm. Complete compensation using robot inverse kinematics. In order to validate the algorithm, a simulation model of the grinding robot with perceivable force information is established. The simulation results demonstrate that the controller trained using this algorithm can achieve constant force grinding without setting up the environment model in advance and has some environmental adaptability.

## Introduction

Traditional manual grinding not only has uneven grinding quality and low efficiency, but also generates flying dust that is hazardous to workers’ health, which can be automated by replacing manual work with robotic grinding [1]. The performance of grinding force is an important factor affecting the grinding quality in the robotic grinding process [2]. Maintaining a constant grinding force during robotic grinding not only ensures the accuracy and consistency of the surface quality of the workpiece, but also reduces the wear and tear on the robot and end-effector.

However, because the robot grinding control algorithm depends on the accuracy of the environmental model and cannot adapt to the environment, etc., constant force control of the grinding process is a challenging problem for robots used in grinding.

The methods of constant force control for robot grinding can be divided into passive constant force control and active constant force control. Passive constant force control is passively adjusted by the passive softening device. Such as a new passive end-effector based on constant-force mechanism for robotic polishing designed by Yuzhang Wei and Qingsong Xu, this new passive end-effector can counteract the force overshoot and improving the force accuracy in the process of polishing [3]; Fan Chen presents a novel two degrees of freedom (DOFs) contact force control method for robotic leaf disc grinding, the grinding tool is controlled to automatically adapt to the curvature change of the leaf disc blade and maintain a constant contact force as expected [4]. However, passive constant force control cannot provide accurate feedback of sensory information and accurate force control. Therefore, active constant force control with perceivable force information has become the focus of research. Robot active force control research has broadly gone through three stages, impedance control, adaptive control and intelligent control. Impedance control was first studied by Hogan and Whitney, and applied to the robot. Impedance control is to add mass-damping-spring system to the end of the robot to establish the relationship between the contact force and the end position. The high-order control barrier functions (HoCBFs) are introduced into the robot impedance control to avoid the violation of time-varying output constraints in Cartesian space by quadratic program (QP). The impedance control is designed to achieve compliance for human–robot interaction (HRI) by Haijing Wang et al [5]. Guanhua Xu et al. proposed impedance-based robot constant force grinding, these methods obtain grinding force through the dynamics model based on the feedback of joint information [6]. Due to the difficulty of parameter identification of the robot dynamics model, the calculation accuracy cannot meet the requirements of grinding. Thus, impedance control does not allow accurate end-effector parameters to be obtained and thus accurate force control is not possible. Adaptive force algorithms change the control parameters of the force controller by altering the contact force status between the robot and the environment, in order to adapt to changes in the environment. Such as Tie Zhang et al. proposed an adaptive iterative constant force control method based on one-dimensional force sensor to improve the processing quality and efficiency of robot belt grinding. Which shows the effectiveness of the proposed force control algorithm [7]. Chang G et al. proposed a constant force control approach to maintain stable force, which is achieved by a position-based impedance control algorithm [8]. However, the adaptive force control algorithm can only adapt to certain disturbances, and the adaptive algorithm is related to the fixed parameters, when the fixed parameters are exceeded, the system will be unstable damage occurs. Therefore, the intelligent force control algorithm becomes the focus of research. Intelligent algorithm of fuzzy control has better robustness, when there is interference or parameter changes in the system, fuzzy control algorithm still has better control effect, so there is better effect in machine human control. For instance, Pokuang Zhou et al. used adaptive fuzzy PID control to grind aircraft engine blades. They proposed adaptive control law to modify controller parameters online, and the grinding force was controlled within 5 ± 0.5 N [9]. Mendes [10] et al. proposed an adaptive fuzzy algorithm and applied it in robot tracking situations, and verified the effectiveness, stability and robustness of this control system by simulation and experimental results, they also designed another fuzzy controller [11]. Other intelligent algorithms like Ting Wang optimized the PID controller using the internal mode control (IMC) principle to control the grinding force using the PID controller [12]. Z. Dachang proposed a PID controller based on fuzzy neural network algorithm for tracking the trajectory and contact constant force simultaneously, numerical simulation results are reported to demonstrate the effectiveness of the proposed method [13]. Jialiang Fan et al proposes a data-driven motion-force control scheme based on the design of a recurrent neural network for the unknown model parameters of the model structure in motion force control [14]. Ubeda, R.P et al used an inner/outer control loop strategy, complementing the robot’s motion control with an outer force control loop to increase productivity [15]. Most traditional intelligent constant force grinding control algorithms are based on environmental models, which means that the environment must be known and predictable, and in some cases, it is impossible to accurately model the environment.

Reinforcement learning algorithms are trained without a prior label of the environment, train and update model parameters by interacting with the environment to obtain feedback rewards from the environment. They compensate for environmental noise by learning even if the environment changes. They have self-learning capabilities, and once trained, reinforcement learning can provide good scheduling results for test data (i.e., data that are not accessible during the training phase) without the need to reformulation and retraining. Reinforcement learning is widely used in various fields. In autonomous driving Hongbo Gao builds the reward function *R* for each driver data based on inverse reinforcement learning algorithm, and performs *R* visualization to analyze driving characteristics and following strategies. Simulations were conducted in a highway environment to verify the effectiveness of the method [16]. A model-based reinforcement learning approach was proposed by Jiren Zhang in an automated parking car to learn parking strategies for data by performing data generation, data evaluation and training the network, and the trained network was used to guide the data generation cycle in subsequent iterations, and the effectiveness was verified through experiments [17]. In robotic grinding, to address the problem that the end contact force is difficult to keep constant when the robot tracks an unknown curved workpiece, Zhang T.et al [18] proposed a machine human control algorithm based on reinforcement learning. For the problem that the parameters of the compensation term are difficult to be selected, the reinforcement learning algorithm A2C is used to find the optimal parameters, and the results show that the optimal parameters found by reinforcement learning can control the grinding force well. The PPO algorithm is an advanced deep reinforcement learning algorithm developed by [19], that can easily handle continuous and multidimensional action spaces; Ming Chen and Chingyao Chan used PPO as a deep reinforcement learning algorithm in autonomous driving and combined with traditional pure tracking methods to build a vehicle controller architecture, and the mixture of the two made the overall system operation more robust and adaptive and improved tracking performance [20]; Bin Huang and Bin Huang proposed a safety control algorithm using a serial strategy in order to strengthen the safety constraints of BSS energy and power capacity, which was used in conjunction with the PPO algorithm and achieved good results [21].

In order to solve the problems of high dependence on the accuracy of environmental model and poor environmental adaptability of traditional control methods, a robot constant force grinding controller that based on proximal policy optimization was proposed. A mechanical model was established to analyze the force of the end-effector; Training the controller model between grinding force difference and end-effector compensation displacement using a proximal policy optimization algorithm, using robot inverse kinematics to complete compensation, including training data normalization pre-processing, Euclidean distance-based reward function design and targeted deep neural network structure design, etc. In order to validate the algorithm, a simulation model of the grinding robot with perceivable force information is established. The simulation results demonstrate that the controller trained using this algorithm can achieve constant force grinding without setting up the environment model in advance and has some environmental adaptability. The following are the innovation points of this article.

1. A robot constant force controller based on Proximal Policy Optimization algorithm is designed, using deep neural network to fit the relationship model between the training grinding force and the end-effector compensation displacement, and completing the compensation using robot inverse kinematics. The completed training deep neural network is used as a robot constant force controller.
2. A simulated robot grinding platform with perceivable force information was built to verify the feasibility and adaptability of the robot constant force controller.

The first section of this paper will present the mechanical modeling of the end-effector, the second section will present the design of the robot grinding constant force controller based on the proximal policy optimization algorithm, the third section will present the construction and experiments of the robot grinding platform that can sense force information, and the fourth section will present the conclusion.

## Mechanical modeling of the robot end-effector

**Fig 1:**
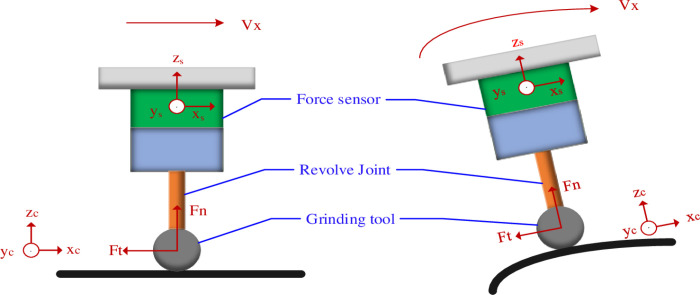
Analysis of end-effector contact forces.

The force analysis of the robot grinding plane and surface end-effector is shown in Fig 1. According to the model during grinding, the positional relationship between the coordinate systems is established: the sensor coordinate system {s}, the workpiece coordinate system {c}: where the origin of the sensor coordinate system is the geometric center of the sensor, the Z-axis direction of the force sensor coordinate system is the axial direction of the sensor, the X-axis direction of the force sensor coordinate system is the radial direction of the sensor, the Y-axis direction of the sensor coordinate system is determined according to the right-hand rule of the coordinate system; the origin of the workpiece coordinate system is the geometric center of the workpiece, the X-axis direction of the workpiece coordinate system is the radial direction of the workpiece, the Z-axis direction of the workpiece coordinate system is the axial direction of the workpiece, the Y-axis direction of the abrasive belt wheel coordinate system is determined according to the right-hand rule of the coordinate system, and the X-axis of the two coordinate systems is kept parallel during grinding.

Let $F_{t}$ and $F_{n}$ be the grinding tangential and normal forces on the workpiece coordinate system, respectively, $F_{t^{\prime }}$ and $F_{n^{\prime }}$ denote the forces that transfer $F_{t}$ and $F_{n}$ to the force sensor coordinate system, then we have $F_{t^{\prime }}=F_{t}$ and $F_{{\text{n}}^{\prime }}=F_{n}$. When grinding operations are carried out using the above positions, the normal force $F_{n}$ is the main object that affects the grinding effect.

As the force sensor is mounted between the end of the robot and the grinding tool, its measured value $F_{s}$ includes not only the normal grinding force at the grinding end $F_{n}$, but also its own gravitational force G and inertial force $F_{l}$, as shown in equation (1), so gravity compensation is performed.


$$F_{s}=F_{n}+G+F_{l}$$ (1)

Since the grinding process is at constant speed and the acceleration is zero, the inertial force Fl is very small and can be ignored. Here the main consideration is the measured value of the sensor for gravity compensation. The grinding robot end-effector is manually adjusted to a vertical downward position without contact with the grinding workpiece, at which point Fn is 0. As the direction of gravity G is always vertical downwards, opposite to the z-axis direction of the base coordinates, it can be expressed as $bFG={\left[0,0,-GT\right]}^{T}$ under the base coordinates. When the robot arm changes position, the value of the base coordinates can be converted to the value of the sensor coordinates using the rotation matrix bsR, i.e. sFG=bsR×bFG, where $sF_{G}$ is the influence value of gravity under the sensor coordinate system. So, the grinding value at the sensor seated $sF_{n}$ is the measured value minus $sF_{G}$, i.e., $sF_{n}=F_{s}-sF_{G}$.

## Design of robot grinding constant force controller based on Proximal Policy Optimization algorithm

Reinforcement learning is performed by the agent as it interacts with the environment, allowing the control strategy to gradually optimize and adapt to the environment. Using deep neural networks, nonlinear problems can be solved, providing a solution for complex control of robot grinding. Among the deep reinforcement learning algorithms, the DQN algorithm was first proposed, but the DQN algorithm can only handle the discrete action space and is not suitable as a continuous action of grinding force control. The DDPG algorithm can handle the continuous action space, but the algorithm itself is difficult to tune the reference. The PPO (Proximal Policy Optimization) algorithm used in this study belongs to model-free on-policy deep reinforcement learning, which can handle continuous state and action spaces and is known for its learning efficiency and usability in various applications.

In this article, agent is the robot and environment are the grinding platform, in the design of the constant force controller, it is necessary to model the relationship between the grinding force difference and the compensation displacement of the robot end-effector. Thus, the grinding force value in sensor coordinates $sF_{n}$ is treated as state.


$$S=sF_{n}=[fx,fy,fz,mx,my,mz]$$ (2)

sFn is the measured value of the force-processed six-dimensional force transducer, which is the force and moment at *x*, *y*, and *z* in the sensor coordinates, respectively. The action is the compensated displacement of the robot end-effector.


A=[dz] (3)

**Fig 2:**
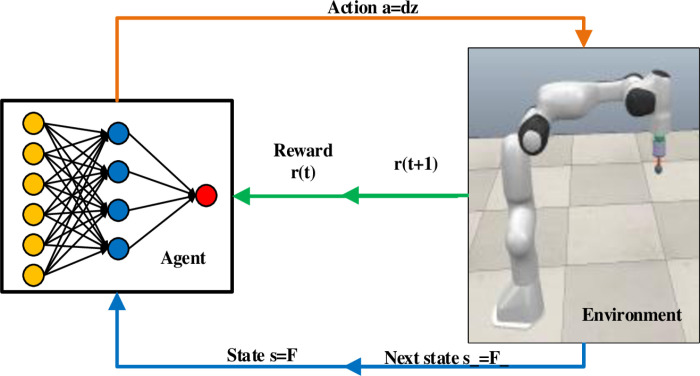
Principle of Grinding Constant Force Control Based on PPO Algorithm.

See Fig 2 for the principle diagram of constant grinding force control based on PPO algorithm.

The future cumulative reward in reinforcement learning is shown in equation (4).


$$G_{t}=R_{t}+\gamma R_{t+1}+...+\gamma^{n-1}Rn$$ (4)

Where γ is the discount factor and takes a value between [0,1].

The PPO algorithm adopts the standard actor-critic framework, the actor uses the method based on the strategy function, and its network outputs the compensation displacement of the end effector after receiving the force information. The critic employs a value function-based approach in which the critic evaluates the actions produced by the actor and gives them recommendations. As the training progresses, the judge improves the prediction accuracy of the reward, and the actor improves the control strategy according to the judge’s suggestion.

**Fig 3:**
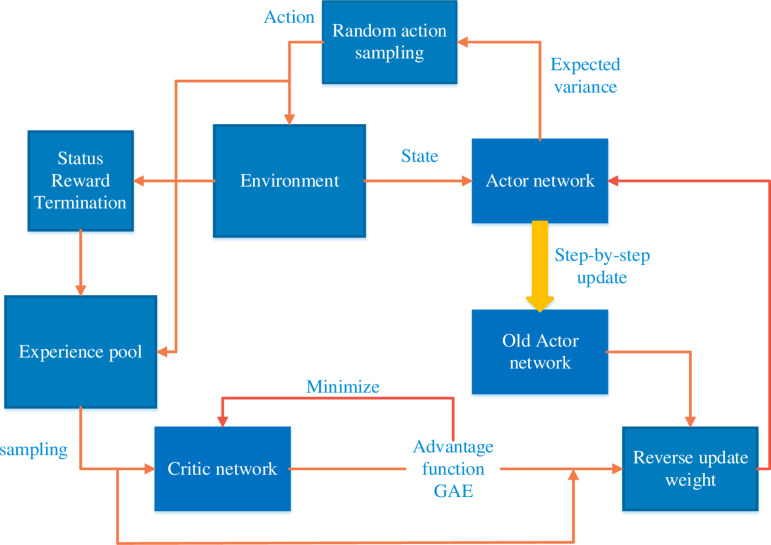
The flow chart of the PPO algorithm.


The flow chart of the PPO algorithm is shown in Fig 3. After receiving the force information, the actor calculates *dz* through the normal distribution formula (5) and completes compensation to obtain the next force information *F_* and reward *R*. The pool stores the system state *F*, action *dz*, reward *R* and the state *F_* at the next moment, and provides data for the actor and critic to use. The actor network produces two output values, mu and Sigma, with mu taking tanh as the value of the final activation function and sigma taking soft-plus as the value of the final activation function. These two values are brought into a normal distribution as the probability distribution of the selected action, i.e., the control policy of the actor.

$$f(x)=\frac{1}{\sigma \sqrt{2\pi }}e^{-\frac{{\left(x-\mu \right)}^{2}}{2\sigma^{2}}}$$ (5)

Critic uses the value of experience pool to calculate the dominance function *A*, and the calculation formula is as shown in (6):


A(F,dz)=TDtarget−V(F) (6)

Where *F* is the state at that moment: force information, dz is the action selected at *F*, *V (F)* is a function of the value of *F*, TD target is the temporal difference (TD) target, and the calculation formula is shown in (7):


$$TD\text{target}=V^{\prime }\gamma +r(t)$$ (7)

Where *γ* is the discount rate and *r* are the reward obtained at time *t*. The obtained dominance function *A* is sent to actor as critic’s suggestion, and the square mean of dominance function *A* is defined as critic’s loss, which reduces the critic’s loss value by back propagation.

In PPO algorithm, some constraints are added to the update of the strategy in order to prevent the difference between the new strategy and the old strategy before the update. The expected reward obtained by the agent adopting the new strategy is compared with the expected reward obtained by adopting the old strategy, and the performance improvement is not expected to exceed a certain threshold, which is called a clipping value and is represented by ε. Since the expected return value of the new policy cannot be calculated when the policy has not been updated, the importance sampling method is introduced, the distribution of the old policy is used to estimate the distribution of the new policy, and the expected return value of the updated policy is calculated as:


$$A^{\pi \theta }(F,dz)=r(t)A^{\pi \theta old}(F,dz)$$ (8)

$$r(t)=\frac{\pi_{\theta }(dz|F)}{\pi_{\theta old}(dz|F)}$$ (9)

The difference between $\pi_{\theta }$ and $\pi_{\theta old}$ should not be too large, and the loss function of the actor is shown in Equation (10).


$$L(\theta )=\min(r(t)A^{\pi \theta }(F,dz),clip(r(t),1-\varepsilon ,1+\varepsilon )A^{\pi \theta }(F,dz))$$ (10)

Where ε is the parameter that ensures that the value of the function is maintained [1−ε,1+ε] in the loss function obtained by updating the actor function with back propagation. The controller design also includes the following.

(1) Data normalization

The input state volume and the output action of the deep neural network are divided by the corresponding upper limit values, respectively, so that each of these elements has a value domain of [1, 1] before entering the algorithm training. The normalized input state volume is denoted as s:


$$s=\left[\frac{fx}{f\max},\frac{fy}{f\max},\frac{fz}{f\max},\frac{mx}{m\max},\frac{my}{m\max},\frac{mz}{m\max}\right]$$ (11)

Where fmax is the maximum grinding force threshold and  mmax is the maximum grinding torque threshold; The normalized output action is denoted as a.

(2) Design of the reward function based on Euclidean distance

The goal of the training is to enable the current grinding force to reach the target grinding force, and the smaller the difference between the current grinding force and the target grinding force, the higher the reward obtained. The reward function used in the design of the constant force controller for robot grinding is the following equation. The European function is used as a reward function because when the force data is closer to the target force, the negative value is smaller, and the reward is larger. When the target force is reached, the maximum reward is obtained, improve the execution speed of the algorithm.


$$r=-\sqrt{{\left(bF-F_{t\mathrm{\backslash arg}et}\right)}^{2}}$$ (12)

Where *r* is the reward value obtained at each moment, bF is the current grinding force and $F_{t\backslash arget}$ is the target grinding force. The obtained rewards are normalized to the same order of magnitude as the input state quantities and output actions into the data used for training.

(3) Designing targeted deep neural network structures

**Fig 4:**
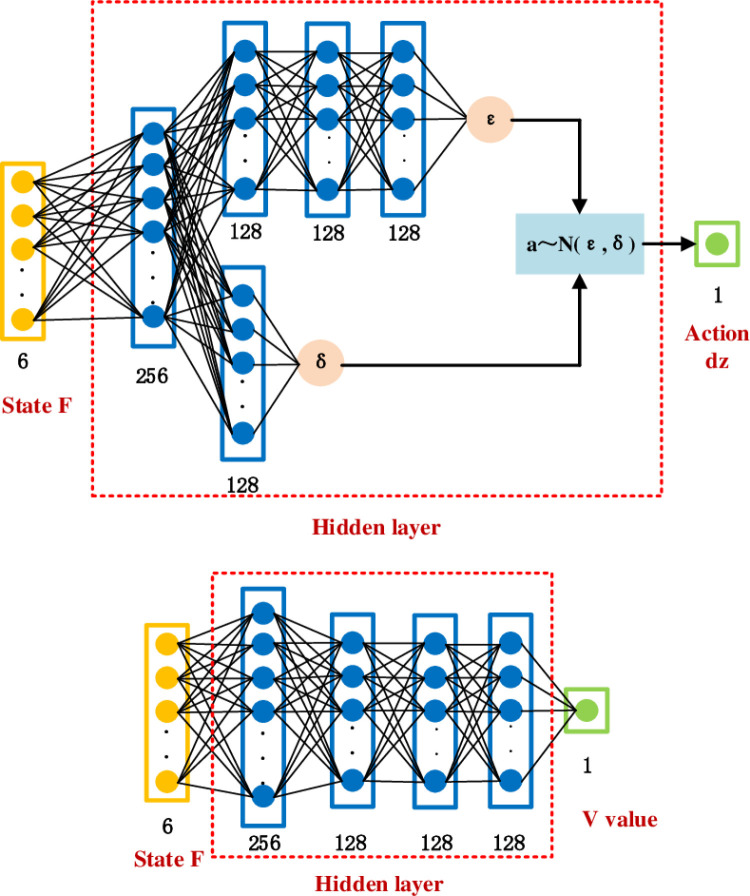
The structure of deep neural network.

Deep neural network structure includes actor network structure and critic network structure. The deep neural network structure is shown in Fig 4.

Actor network structure for six layers, including the input layer, hidden layer and output layer, the hidden layer has four layers of nodes in the order of 256, 128, 128, 128, choose Rule activation function.

The critic network structure calculates the mean and variance of the outputs, denoted ε and δ, respectively. The neural network includes six hidden layers, which are input layer, hidden layer and output layer. The number of nodes in hidden layer is 256, 128, 128 and 128 respectively. The neural network used to calculate the output variance includes two hidden layers, and the number of nodes is 256 and 128 respectively. The first hidden layer of the output mean value calculation part and the first hidden layer of the output variance calculation part are the same network structure layer, the activation functions among all the hidden layers of the critic network structure are Relu activation functions, the activation function before the output layer for calculating the output mean value ε is a Tanh activation function, the activation function before calculating the output variance δ is a Soft-plus activation function, and the output of the strategy network structure is a sampling value of Gaussian distribution.


a~N(ε,δ) (13)

In this article, Pseudocode for the proximal policy optimization-clip algorithm is shown in Table 1.

**Table 1:** Pseudocode for the proximal policy optimization-clip algorithm.

Algorithm
1: Input: Initialize the policy parametersθ and the value function parametersφ
2: Start the cycle: K=1,2,…,N
3: The intelligence interacts with the environment using the current moment's strategy$\pi_{\theta k}$ to form a set of M trajectories$D_{k}=\{\tau_{i}\}$ , each containing a finite number of time steps T
4: Calculate the approximate value of each moment of return for each trajectory using the TD method${\hat{R}}_{t}$
5: Calculate an approximation to the dominance function${\hat{A}}_{t}$ based on the current value function$V_{\phi k}$
6: Maximize PPO-clip targets to update policy parameters $\theta_{k+1}=\underset{\theta }{\mathrm{\backslash arg}\text{ max}}\;\frac{1}{N}\sum_{\tau \in Dk}[\frac{1}{T}\sum_{t=0}^{T}\min[L(\theta )]$ $L(\theta )=\min[r(t)A^{\pi \theta k}(s,a),clip(r(t),1-\varepsilon ,1+\varepsilon )A^{\pi \theta k}(s,\;a)]$
7: Updating the value function parameters using regression to the mean of the squared error and gradient descent
8: $\phi_{k+1}=\underset{\phi }{\mathrm{\backslash arg}\text{min}}\;\frac{1}{M}\sum_{\tau \in Dk}[\frac{1}{T}\sum_{t=0}^{T}{\left[V_{\phi }(s_{t})-{\hat{R}}_{t}\right]}^{2}]$
9: End

## Experiment

Coppeliasim is selected as the simulation software platform. Coppeliasim not only contains many encapsulated robots for easy use, but also supports Bullet and other physical engines. The physical engines enable the established simulation model to have mechanical properties and more accurately simulate the real environment. At the same time, Coppeliasim includes a remote API that allows communication with languages such as Python, solving the problem that the Lua language built into Coppeliasim cannot build neural networks and enabling co-simulation. In addition, Coppeliasim provides an inverse kinematics solution module based on the numerical iterative solution, which can realize the function of directly outputting and executing the control action of the end tool pose by the grinding force control algorithm.

**Fig 5:**
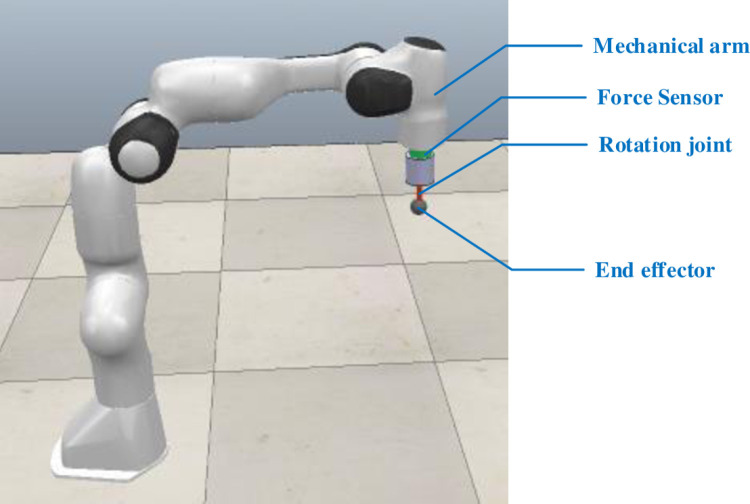
Force-aware robot grinding simulation model.

The force-aware robotic grinding simulation platform consists of a robot, a six-dimensional force sensor, a rotary axis, an end grinding actuator and the workpiece to be ground. The robot is packaged with the Franka robot in the simulation platform and the six-dimensional force sensor is mounted at the end flange of the robot to obtain the grinding forces and grinding moments in the *x*, *y* and *z* axes of the sensor. As the end grinding actuator component does not exist in the simulation software, it can be modelled using drawing software, imported into Coppeliasim to set the mechanical characteristics and set the rotary axis to drive the end grinding tool for grinding operations. The robot end flange, the six-dimensional force sensor and the end grinding actuator need to be in the same *Z* coordinate. The force-aware robot grinding simulation model is shown in Fig 5.

**Fig 6:**
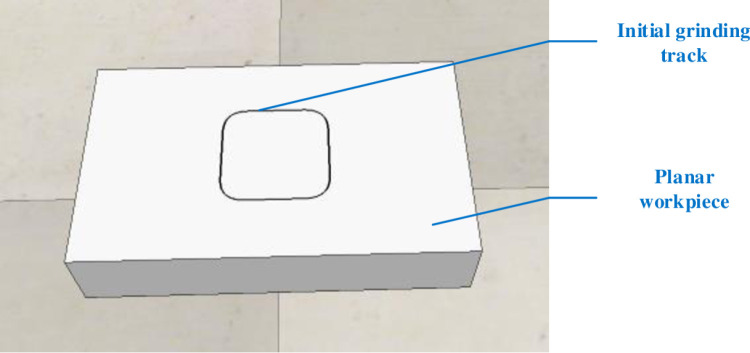
Planar workpiece and initial grinding trajectory.

Import the planar workpiece into Coppeliasim to set the attributes, set the grinding initial trajectory by teaching, and let the robot end move along the initial trajectory. The planar workpiece and the initial trajectory are shown in Fig 6.

**Fig 7:**
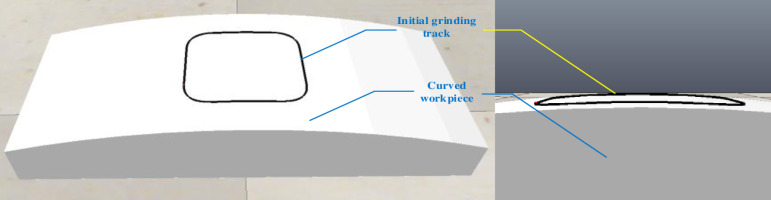
Surface workpiece and grinding initial trajectory.

The curved surface workpiece is imported into the simulation software in the same way, and the initial grinding trajectory is drawn through teaching as shown in Fig 7.

**Fig 8:**
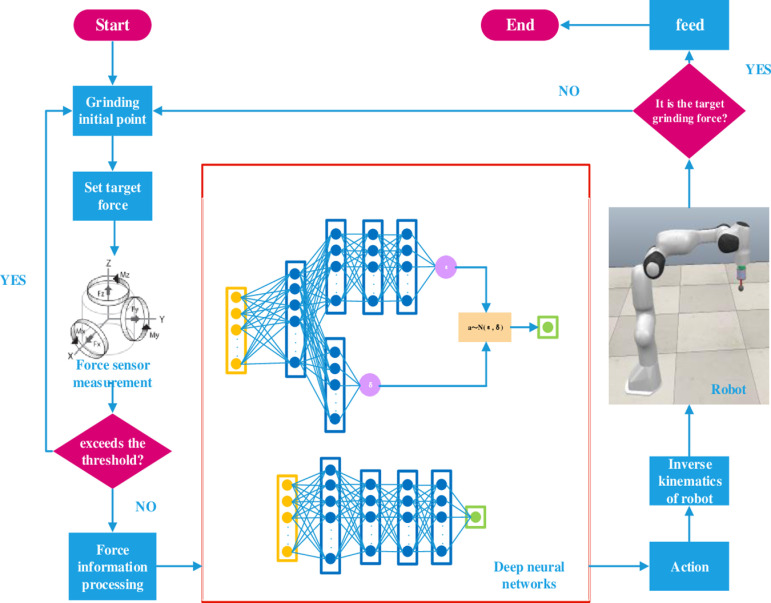
The flowchart of grinding constant force control based on the PPO algorithm.

This paper only studies the grinding process, that is, before the realization starts, the end effector of the grinding robot is manually moved above the starting point of the grinding workpiece, so that the end effector contacts the grinding workpiece, and the six-dimensional sensor obtains the initial force information, so as to carry out the training experiment. The flowchart of grinding constant force control based on the PPO algorithm is shown in Fig 8.

The PPO algorithm is used for robot grinding constant force control training. The total number of training sessions is set to 100, and each training session consists of 200 training steps, in which the robot and the workpiece interact, and the data from the interaction are stored in the experience pool according to the time series regardless of the control effect. The trained model is required to satisfy the convergence of the deep network structure to a stable state.

In order to make the controller more adaptable to the environment and accept more force information, the initial normal position of the end-effector is reset after one training session, and the value within the initial position ± 0.01m interval is randomly selected as the grinding starting point for a new training session.

The trained neural network structure is used as a controller for the polishing constant force control of the robot, the input is the normalized force information F, and the output is the normalized compensation displacement dz of the end effector. And that control position of the robot is generated after bee added with the normal coordinate of the current end-effector, the joint angle of the robot is obtained through the inverse kinematics of the robot to control and realize compensation, and the whole process of designing, training and verifying the controller is completed in a simulation environment. The weight of the end effector is set as 0.3 kg, and the specific neural network parameters of the PPO algorithm are shown in Table 2.

**Table 2:** Neural network parameters.

Parameter	Value
Discount factor(γ)	0.9
Learning rate Actor	0.0001
Learning rate Critic	0.0003
Batch	32
Epsilon(ε )	0.2
Clipping	0.2

The controller is applied to the following two cases: robot grinding plane workpiece and robot grinding curved workpiece to verify the feasibility and adaptability of the robot grinding constant force controller based on PPO algorithm.

1. Constant Force Control of Robot Grinding Planar Workpiece

**Fig 9:**
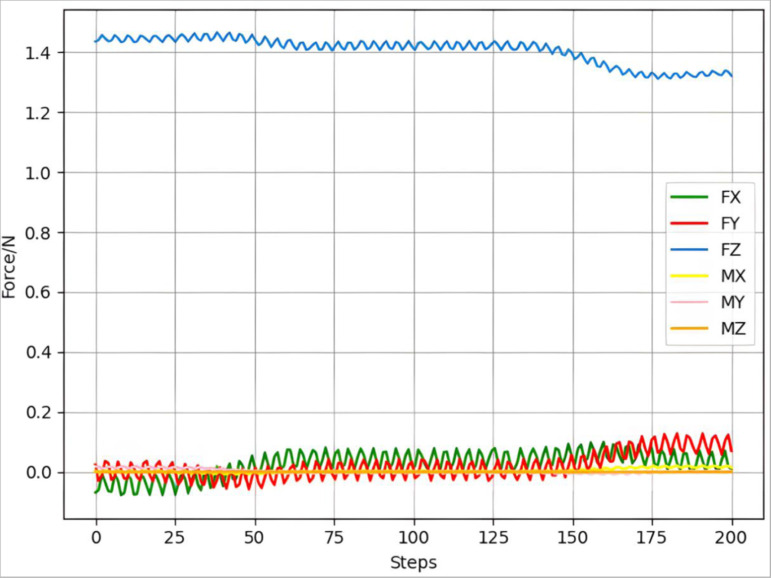
Six-dimensional force change without constant force controller.

First, grinding was performed without using a constant force controller, and a six-dimensional force change diagram is shown in Fig 9. It can be seen from the figure that without the constant force controller, the value of the six-dimensional force, especially the normal force Fz is unstable. Unable to meet grinding requirements, force control is required.

**Fig 10:**
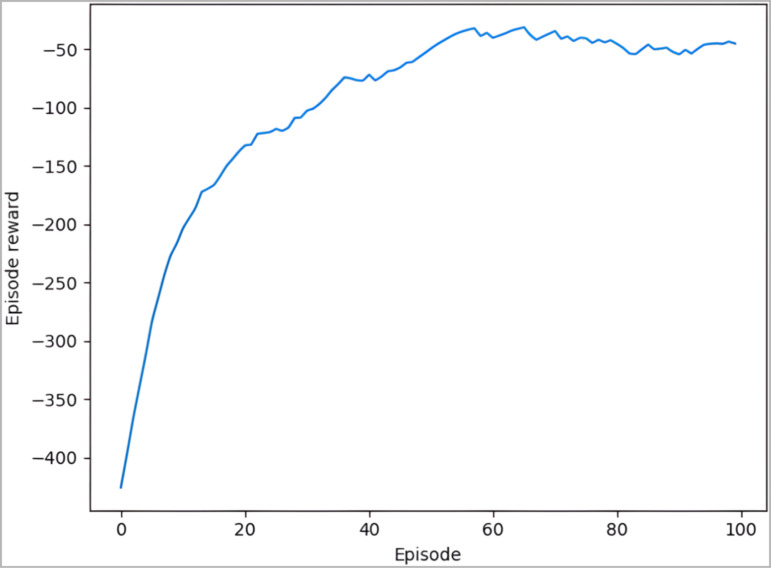
The total reward diagram for the constant force control of the robot grinding planar.

The force controller training method is performed as shown in Fig 8, and the force controller is stored after the deep neural network converges and used as a grinding constant force controller. The total reward diagram for the constant force control of the robot grinding planar obtained at the end of the training is shown in Fig 10. It can be seen from the figure that with the increase of training times, the total reward value increases continuously, and finally stabilizes around -50. It shows that the gap between the current grinding force and the target grinding force is narrowing, and the current grinding force is approaching the target grinding force, and finally fluctuates above and below the target grinding force. It shows that the trained controller model has converged to a stable state.

**Fig 11:**
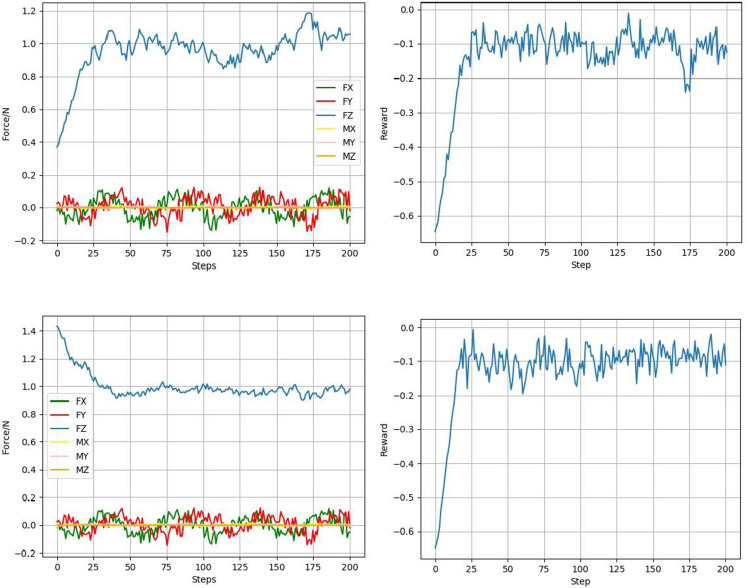
The six-dimensional grinding force change and reward function change.

After the convergence of two groups of experimental results, the six-dimensional grinding force change and reward function change of the two groups of experimental results after convergence are shown in Fig 11. It can be seen from the figure that the initial grinding forces are different because the normal positions of the initial end effectors are different, but whether the initial grinding forces are less than or greater than the preset grinding force, the controller can make the current grinding force quickly close to the target grinding force and fluctuate on the predetermined grinding force, thus realizing the constant force control of a planar workpiece.

2. Constant Force Control of Robot Grinding Curved Workpiece

**Fig 12:**
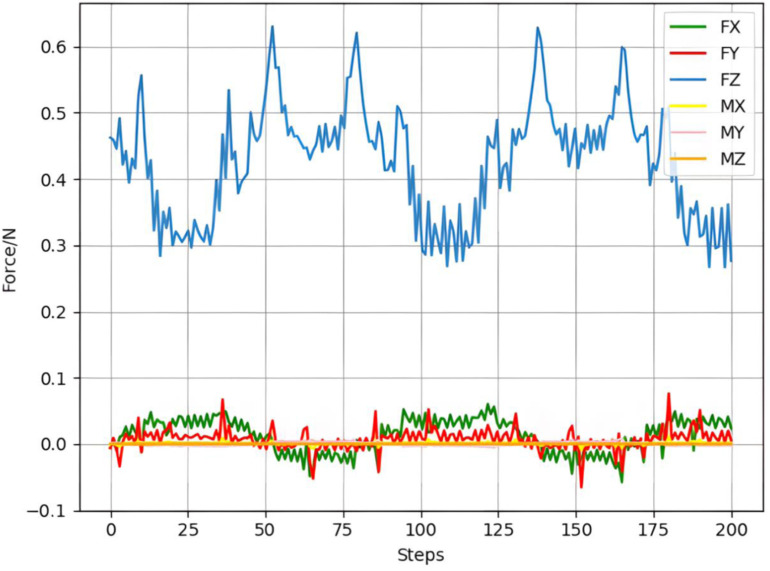
Six-dimensional force change without constant force controller.

The curved surface grinding is carried out according to the same steps as the surface grinding. First, the grinding is carried out without using the constant grinding force controller. The six-dimensional grinding force change diagram is shown in Fig 12.

**Fig 13:**
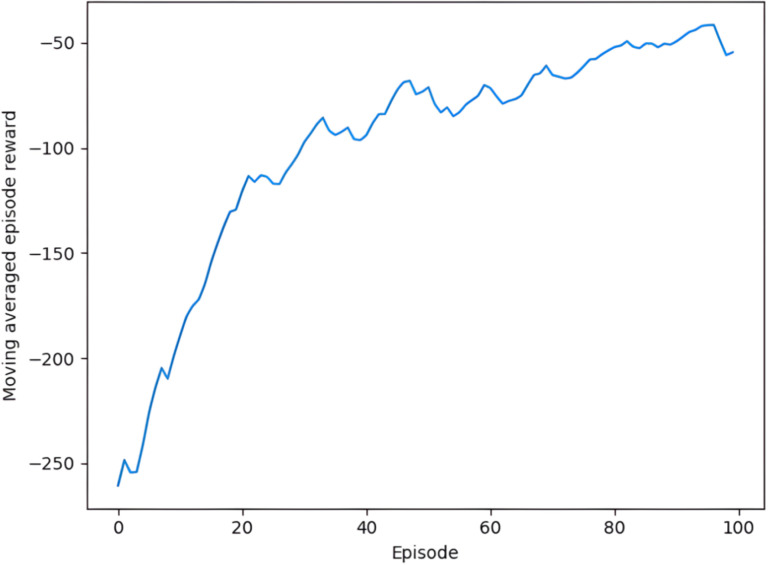
The total reward diagram for the constant force control of the robot grinding curved.

It can be seen that the grinding force is very unstable when grinding curved surfaces (0.3N-0.6N), and constant force control is needed. The force controller training method is performed as shown in Fig 8, and the force controller is stored after the deep neural network converges and used as a grinding constant force controller. The total reward diagram for the constant force control of the robot grinding curved obtained at the end of the training is shown in Fig 13.

It can be seen from the figure that although the total reward curve for planar grinding does not converge as well as for flat grinding, the total reward value increases with the number of training sessions and finally tends to fluctuate around -50. It shows that the gap between the current grinding force and the target grinding force is also narrowing, and finally the target grinding force fluctuates.

**Fig 14:**
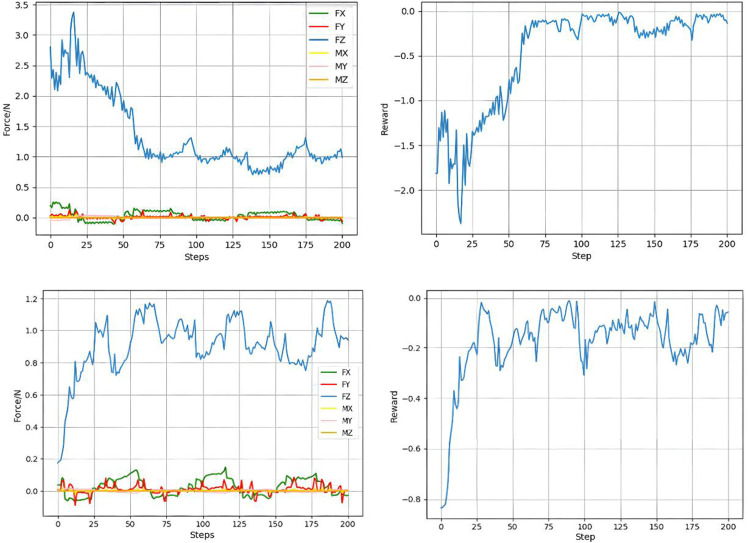
Variation of six-dimensional grinding force and reward function.

As in the case of surface grinding, after the controller converges, the results of two experiments are taken out, and the six-dimensional grinding force and reward function variation diagram is shown in Fig 14. It can be seen from the figure that although the initial grinding force is different, after the grinding constant force controller is used, the grinding force continuously approaches the target grinding force, and finally fluctuates continuously within the range of the target grinding force ±  0.2N. The effectiveness of constant force control for grinding curved surface is illustrated.

Due to the complexity of the actual grinding environment, before the controller is directly used in the actual situation, the end effector is contacted with the polished part, and the grinding experiment is carried out after the force data is maintained at the target grinding force to prevent damage to the robot. The fluctuation of the actual force data will be greater than the simulation result, but constant force grinding can still be carried out.

## Conclusion

A robot grinding constant force controller based on PPO (Proximal Policy Optimization) algorithm is designed. The method solves the problems that the traditional robot force control method has high dependence on the accuracy of an environment model and poor environmental adaptability. In order to verify that algorithm, a robot grinding simulation model capable of sense force information is built by using Coppeliasim, two simulation experiments of a plane and a curved surface are carried out, and the simulation experiments prove that the constant force controller can keep constant force in the plane and the curved surface grinding, have certain environmental adaptability. The algorithm proposed in this paper cannot guarantee the real-time performance accurately in practice, so the research on strengthening the real-time performance of the algorithm will be carried out in the future

## Supporting information

S1 Data Minimal data set. (ZIP)
